# Social Sciences in One Health: Insights From Multiple Worlds Perspectives on the Dam Rupture in Brumadinho-Brazil

**DOI:** 10.3389/fpubh.2021.649355

**Published:** 2021-09-29

**Authors:** Ana Pérola Drulla Brandão, Stefanie Sussai, Jéssica Alves de Lima Germine, Diego Duarte Eltz, Aline Araújo

**Affiliations:** ^1^Department of Preventive Medicine, Faculty of Medicine, University of São Paulo, São Paulo, Brazil; ^2^Department of Preventive Veterinary Medicine and Animal Health, Faculty of Veterinary Medicine and Animal Science, University of São Paulo, São Paulo, Brazil; ^3^Graduate Program in Human and Social Sciences, Federal University of ABC, Santo André, Brazil; ^4^Graduate Program in Social Anthropology, Federal University of Rio Grande do Sul, Porto Alegre, Brazil; ^5^Graduate Program in Social Sciences, University of Vale do Rio dos Sinos, São Leopoldo, Brazil

**Keywords:** alternative economy, equivocations, extractivism, Indigenous worlds, one health, perspectivism, pluriverse, transdisciplinarity

## Abstract

Concepts that integrate human, animal, and ecosystem health - such as One Health (OH) - have been highlighted in recent years and mobilized in transdisciplinary approaches. However, there is a lack of input from the social sciences in OH discussions. This is a gap to overcome, including in Latin America. Therefore, this paper incorporates recent studies from economics and anthropology to the debate, contributing to the opening of transdisciplinary dialogues for the elaboration of OH theory and practice. As a starting point, we explore the recent case of a tailings dam breach, making considerations about how and why this event was experienced in different ways by the affected Indigenous and non-Indigenous worlds. From economics, we show how different theories perceive and impact these different worlds, presenting some existing alternatives to the hegemonic thinking of domination and exploitation. From anthropology, we present the perspectivism concept, deriving from the field of relational ontologies, suggesting there are significant and inevitable disagreements-equivocations-among different worlds. Thus, we discuss how the social sciences can help address challenging factors that need to be considered in health approaches that intend to deal with complex global problems. In conclusion, OH should incorporate social science discussions, considering relating practice to the multiple realities in which a particular problem or conflict is inserted. Overcoming the barriers that hinder transdisciplinary dialogue is fundamental and urgent for an effective approach to the multiple and distinct interconnections among humans, animals and environments.

## Introduction

In recent years, some holistic health perspectives such as One Health, EcoHealth, and Planetary Health have grown in importance, and their concepts have undergone a process of constant refinement. Some differences between these terms have been studied and described, such as their origin and central focus, the sciences contributing to each of them, and how they value humans, animals and ecosystems (1). However, despite their differences, a common aspiration is toward integrative, collaborative, transdisciplinary, and multisectoral approaches that acknowledge the health of people, animals and the environment as “one” (2–7).

In common, such concepts also express a disagreement with the traditional Western thinking that radically separates and opposes nature and humanity. Thus, pairs of opposites like nature/humanity - among others derived from it, such as physical/metaphysical, objective/subjective, humanity/animality - has underpinned the way Western thinking understands nature (including animals) and relates to it, also reflecting how health and disease are differentiated. Therefore, the modern world winds up limiting entire ecosystems to an object, a resource to be controlled and managed to satisfy human needs.

This way of thinking led to what is conventionally called the *Anthropocene* (See **Panel 1** for the glossary of terms used in this paper) (8). One of the factors that characterizes this new geological era is the expansion of mineral, oil and biotechnological extraction. These large-scale extractive activities have harmful impacts that are not homogeneously produced or distributed among the different strata of society. Besides, such impacts affect the health of people, animals and ecosystems (9, 10). Therefore, the effects of the Anthropocene on the planet are an issue to be considered by integrative health discussions (5).

Considering the holistic health perspectives, health and disease are no longer understood solely as qualities or conditions of an isolated individual - whether human or not - but rather, of a multispecies collective living in the Anthropocene epoch. From this assertion, it is clear that the social sciences can contribute to such health debates, since they focus on the interplay of humans, society, and nature.

Focusing on the *One Health* concept **(Panel 1)**, early aspirations about the potential to address both social and ecological concerns have made landmarks and driven following studies since (2, 11). The significant role but also the underrepresentation of the social sciences have been described in recent years (12–14). Despite some initiatives, such as the efforts of the One Health Commission (15), this lack is still significant. Particularly in Brazil, the scientific publications using the term One Health have been mostly limited to the sphere of veterinary science and public health. This fact, which we consider to be a problem, is certainly multi-causal, but is also due to the strength of the disciplinary divisions that configure the scientific practices in the country.

With the paucity of social science perspectives within the One Health space, this current article therefore aims to contribute to the opening of *transdisciplinary* dialogues **(Panel 1)** in the elaboration of One Health theory and practice in Brazil - and in other Latin American countries that could benefit from this integration. Utilizing a particular event as an example and starting point, we present *challenging factors* **(Panel 1)** from studies and recent discussions in economics and anthropology that exemplify the need for transdisciplinary discourse.

## Starting Point: Tailings Dam Rupture in Brumadinho, Minas Gerais State, Brazil

**Figure 1:**
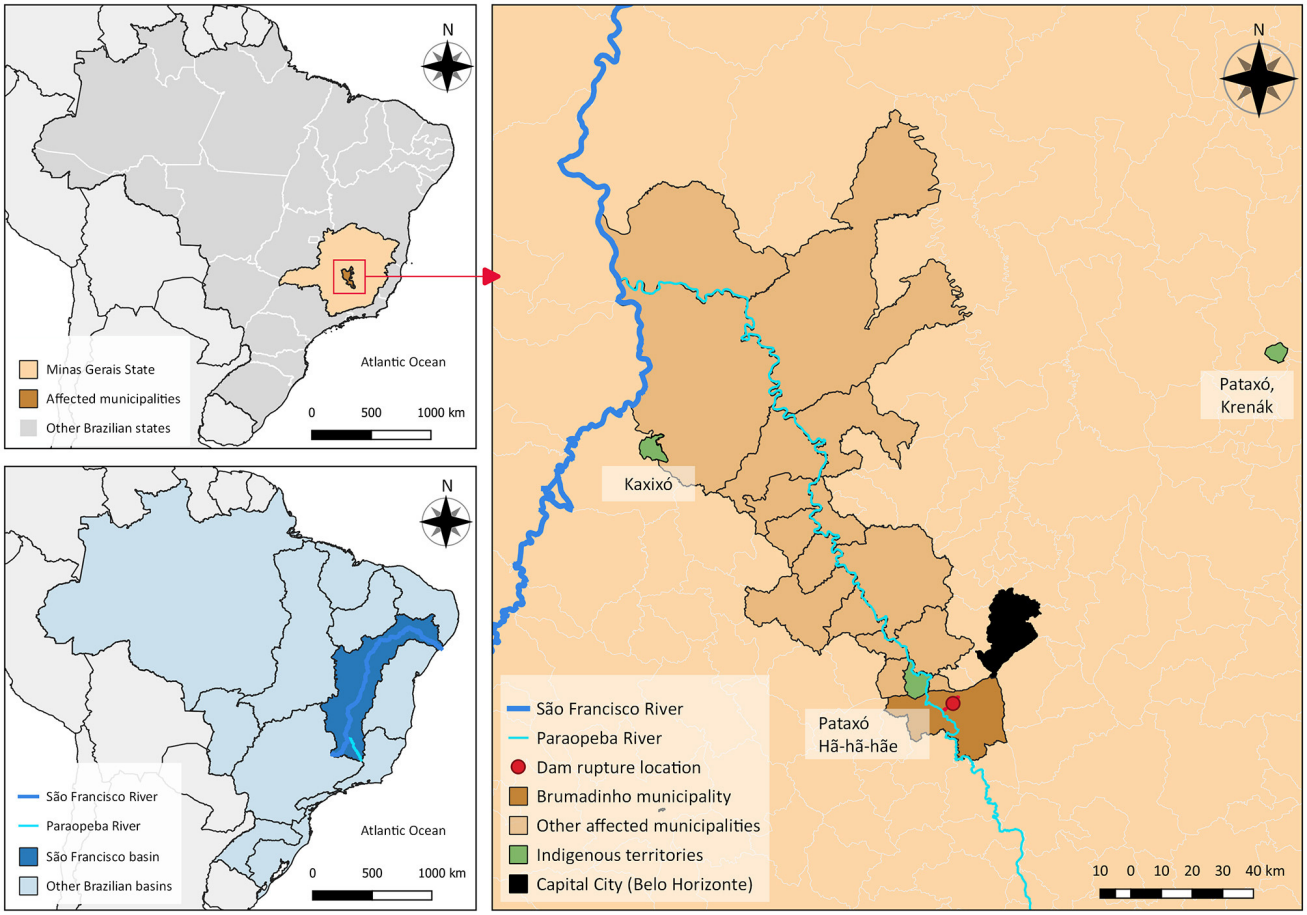
Location of Brumadinho dam rupture, showing the extension of the impact on rivers, municipalities, and Indigenous territories.

In January 2019, a tailings dam operated by Vale S.A., the world's largest iron ore producer (16), collapsed in the municipality of Brumadinho. Nearly 13 million m^3^ of iron ore tailings (17) reached the tributaries of the Paraopeba River, part of the São Francisco Basin: one of the main watersheds in the country. The toxic mud traveled along the river, causing irreversible ecosystem damages, and affecting several other municipalities, including Indigenous territories such as the Pataxó Hã-hã-hãe and Kaxixó (18) (Figure 1). A total of 259 people died and 11 are still missing and assumed dead (19). The impacts of the tragedy are certainly far-reaching and long-lasting, and the socio-environmental damages are systemic, synergistic, and dynamic, involving the health, environment, economics and rights of people, animals and affected areas (20, 21).

This tragedy occurred just 3 years after a similar one in the same region, in the municipality of Mariana, when another dam-co-owned by Vale-released 45 million m^3^ of iron ore tailings, reaching the tributaries of the Doce River and then the Atlantic Ocean. Since then, the company was aware of the risk of failure of the dam in Brumadinho, which means that the disaster was not natural and could have been avoided (17).

From this context and its consequences, we bring for reflection two very different statements about what happened. The first came from Vale's CEO: “Vale is a Brazilian jewel that cannot be condemned for an accident that happened in one of its dams, no matter how great its tragedy may have been” (22). The second one came from the Chief of the Naô Xohã village, where 25 Pataxó Huh-hã-hãe families lived: “It was a funeral without a wake. A piece of our body was cut off” (23).

These statements exemplify how and why events like this can be experienced and understood in different ways by the Indigenous and non-Indigenous worlds. For the mining company and the Brazilian government, the damage to the Paraopeba River represents an *externality* **(Panel 1)** that cannot compromise development. For the Pataxó Hã-hã-hãe Indigenous people, the river is not only what Western thinking understands as nature, but also a part of themselves - the watercourse is also a life course. Based on this context as a starting point, this paper relies on theories and discussions of economics and anthropology to suggest there are significant disagreements among *multiple worlds* **(Panel 1)**.

## Economics: How Different Theories Perceive and Impact Multiple Worlds

In the statement by Vale's CEO, expressions such as “accident” and “cannot be punished,” refer to the hegemonic economic thinking that treats environmental impacts as negative externalities. In this respect, externality refers to “side effects” arising from productive or consumption actions (24), that is, factors external to the system (25). In this case, the private benefits of the company's activities, measured in monetary terms, were prioritized to the detriment of the socio-environmental costs of the dam rupture, which cannot be precisely quantified.

In this productive system, the notion that “a river is a water pipe and animals are protein factories” (26) has become institutionalized. Since private property is a key element, the *common goods*- those shared by everyone and that do not belong to an individual or group, such as what is called natural resources-can be 'managed' and tend to be overused, generating a negative externality to the environment or society.

For Vale, as well as for the entire economy derived from classical and neoclassical theories, the workable solutions offered to minimize such effects are limited to the creation of taxes and subsidies or some kind of “externality market.” Then, in theory individuals can negotiate the costs derived from their activities (25, 27). Therefore, it is common for the mining, agriculture and tourism sectors to measure their impacts in monetary terms. However, it is impossible to attribute value to the lost lives, or to the socio-environmental impact caused - including the death of an entire river.

In this sense, two phenomena create and intensify the disastrous ecological scenario. The first is the dual and hierarchical perception of the world (such as human and nature). This is a way of justifying and legitimizing relations of domination, whether among humans or between humans and other-than-humans. The second is the fictions derived from traditional economics and imposed as absolute truths. These fictions are responsible for legitimizing and establishing economic fundamentalism as hegemonic, such as the idea that production is unrelated to life (26).

Alternative economic theories have emerged in opposition to the traditional ones and can enrich the dialogues with One Health since they question the idea that nature is just an object or resource. The *political ecology* focuses on socio-environmental conflicts, proposing the integration of indicators to broaden the view of the consequences of economic development in different populations and territories (28). The *ecological economy* contests the meaning of development and its implications, going beyond the concept of sustainable development and proposing an alternative to it. In addition, it presents multicriterial strategies allied with environmental policies to deal with such effects, such as the so-called externalities (29). The *theories of degrowth* presuppose a break with the production and consumption system based on capitalist domination and exploitation, through self-limitation and moderation (30), and abandonment of unlimited economic growth (31).

In this direction, we cannot fail to mention the idea of *buen vivir*- good living - born in Latin America and influenced mainly by Andean and Amazonian Indigenous roots. Buen vivir is a plural concept conceived by the confluence of theoretical debates, Indigenous practices, social movements, and political constructions (32). Also, buen vivir questions the concept of well-being based on Eurocentric assumptions, defends overcoming the idea of development as a synonym for material accumulation, and offers alternatives to it (33, 34). The principles of buen vivir were formalized in the new Constitutions of Ecuador and Bolivia, as a fundamental base of the State.

Therefore, the alternative to the current scenario would be to overcome dualisms, admitting eco-dependence and interdependence, as well as placing life at the center of economics and politics (26), in order to build a possible non-domination system. In this sense, we introduce and explore a field of anthropology called *relational ontologies* **(Panel 1)**, which shows that the dualistic ontology (that radically separates nature from humanity), despite its universal claim, is not the only one (35).

## Anthropology: When Assumptions are not Common - or the Same - Among Worlds

The studies of relational ontologies concern the interrelationships of a broad community - considering community as a concept that “initially human-centered, is expanded to include other-than-humans” (35). Situating the practices of modernity in space and time, the relational ontologies demonstrate that not all worlds are made from the same divisions, such as human/other-than-human or culture/nature.

One example of relational ontology studies is *perspectivism*, formulated by Viveiros de Castro in 1996 (36). It has become one of the most cited concepts in Brazilian anthropology, and also is the most notable theoretical contribution to global anthropology (37). The term “perspectivism” comes from philosophy and was borrowed to highlight a striking aspect of Amerindian worlds: the way human beings see animals and other subjectivities is profoundly different from the way these beings see humans and themselves.

The notion of other-than-human beings having their own perspective comes from a great mythical division (36, 38, 39) that is “shared by several, if not all, Indigenous people of the New World,” as is stated by Viveiros de Castro (40). According to de Castro (41), unlike the Western evolutionary vulgate - which uses soul and, more recently, consciousness or culture as criteria to distinguish humanity from animality - the Amerindian perspectivism states that the original condition of other-than-human beings is humanity, not animality. Their bodies, as we see them, are clothes that hide their internal human form, which is only visible to those of the same species or trans-specific beings, such as shamans. Thus, back in their homes - as the humans they are - they hunt, fish, fight, and perform rituals. If we start to see from their perspective, it means that our soul has been stolen or that we are being taken to a different world (41).^1^

The perspectivism discussion confronts the modernistic idea that there is only one shared world - one external and objective reality - and multiple representations of it, i.e., worldviews or cultures. The modern way of thinking enables cultures to be hierarchized according to how distant their representations are from that one reality. Such hierarchy allows a specific culture to have the privilege and monopoly of defining terms such as nature, culture, humanity, animality, health and disease (42). Instead, the perspectivism points to a *pluriverse*- multiple worlds that share the same culture, and even use the same terms, but differ according to the perspective of the referent, whether human or other-than-human. Thus, there is no privileged perspective to define reality.

Since these multiple worlds are not based on the same assumptions and divisions, there may be significant disagreements between them. In that respect, a category of perspectivism arises called *equivocations* **(Panel 1)**, which emerges when different worlds use the same term to refer to different things. Because these equivocations are a result of a communicative relationship between different worlds, they express an ontological relationship and not an epistemic misconception (43).

We believe the Brumadinho catastrophe can illustrate an equivocation. As previously exemplified, for the Indigenous world, the Paraopeba River was a life course. This *uncommon*^2^ status of the river is unacceptable for the modern world. For Vale and the Brazilian State, a river is not - and cannot be - different from a hydrographic formation, a formless universally shared *common good* that can be managed and exploited as an externality (44).

Since equivocations emanate from different worlds, they cannot be avoided. However, they can be controlled^3^ by a communicative exercise that considers the referential otherness of the different perspectives, maintaining and communicating their ontological differences (45, 46). This exercise invites us to think of a *common alternative*, namely “the expression of an ecology of divergent practices, constantly negotiating what would be their common interest” (44). Therefore, we suggest that identifying equivocations and being open to this communicative exercise can be a key element in any integrative health approach.

## Discussion

One Health professionals and researchers often address complex health phenomena and recognize the importance and need of integrating different fields of knowledge. The collaboration among disciplines can be imagined and carried out in diverse ways and with different objectives, as shown by the concepts of multi-, inter-, meta-, pluri- and transdisciplinarity (47–49). The last (and most complex) seeks knowledge *between, through*, and *beyond* disciplines, without a hierarchical relationship among them (50).

In Brazil, transdisciplinarity in health has advanced since the 1970's, with two historical movements that emerged in the context of fighting for democracy and against the military dictatorship that lasted from 1964 to 1985. The first was the Brazilian Health Reform, resulting in the creation of the Unified Health System [SUS (Portuguese acronym)] (51). The second was the political-ideological-intellectual movement of Collective Health, resulting in a whole new field of health studies and practices (52). In the 1990's and 2000's, transdisciplinarity became more widespread after the SUS implemented its Family Health Strategy, with multi-professional teams working to promote health beyond the hospital environment (53).

However, in Brazil there are still many barriers that hinder knowledge sharing and unification, such as historical institutional structures, values and habits (54, 55). These obstacles, imposed by modernity, can manifest themselves as “social, pedagogical, ideological, political, psychological, methodological and technical” (54). Therefore, overcoming these barriers is a challenge additionally for professionals and researchers who seek to act within the realm of One Health.

Our starting point - the analysis of the dam rupture - provided elements that relate to some recent discussions in economics and anthropology. Such discussions are an example of the undeniable contribution of the social sciences to One Health issues, since they highlighted the existence of *challenging factors*- such as huge environmental impacts considered as mere externalities and the existence of equivocations between different worlds. These challenging factors need to be seriously considered by health approaches that intend to be integrative, since they increase the awareness of the complexity of health topics.

It is important to point out that the Brumadinho disaster is not an isolated event in Latin America. Other examples, just to cite a few, are the continuous oil spills in Ecuador (56–58), environmental impacts of transgenics in Argentina (59), and disasters caused by mining in Chile (60). Such events and how they are usually managed show that the assumption that there is a passive, sacrificial and appropriate nature promotes huge pressure and impact on people, animals and ecosystems - especially on those in situations of vulnerability and living in countries with high social inequality (61).

Besides, the assumption that nature is an object to be sacrificed for human interests and needs reinforce and reiterate asymmetries, producing *regimes of truth* **(Panel 1)** and invalidations, that is, relegates other perspectives to a status of mere beliefs or metaphors (42). However, such assumption is neither natural nor cosmopolitan: it comes across the borders of other worlds, such as the Indigenous ones, which refuse to obey the mandate of the nature/humanity division and resist the imposed extractive projects (44). The point is: the communicative exercise between worlds is important to make sure that no regime of truth is reproduced and no world is neglected in the process of decision making on health problems that concern multiple worlds.

Since One Health is proposed to be transdisciplinary and approach increasingly complex global health challenges, its practice and scientific production should not reproduce regimes of truth and invalidations. On the contrary, One Health should be open to the idea that the multiplicity of interactions among humans, animals and ecosystems can be formed by different assumptions, linked not to cultural differences, but ontological ones. Therefore, One Health professionals and researchers should be aware of - and closer to - discussions of alternative economic theories along with the perspectivism and the debate of multiple worlds - especially those that conduct research in Latin America, due to the ongoing impact of the extractivism previously discussed. Thus, people involved in One Health can facilitate and participate in transdisciplinary dialogues, overcoming the disciplinary barriers that divide the scientific practices in their countries.

## Conclusion

Considering the challenging factors exemplified by the Brumadinho dam failure under the economics and anthropology lenses, we suggest that to achieve their goals, researchers and practitioners using One Health approaches should incorporate discussions of alternative economic theories and the multiple worlds perspective. This would help to reduce the limited dualistic and anthropocentric views and regimes of truth. Moreover, we argue that One Health should always be related to the context of the realities - plural - in which a particular problem, conflict or challenge is inserted. This implies that One Health should be plural or have several versions.

Based on the discussion of extractivism, transdisciplinarity and contributions of the social sciences, we suggest that in Brazil - and other Latin American countries with a similar context - it is fundamental and urgent to overcome disciplinary barriers in One Health. That is, it is essential to include the social sciences and their professionals in One Health debates, for an effectively transdisciplinary dialogue about the multiple and distinct interconnections among humans, animals and ecosystems.

## Data Availability Statement

The original contributions presented in the study are included in the article/supplementary material, further inquiries can be directed to the corresponding authors.

## Author Contributions

AB and AA conceptualized the paper. AB and SS contributed to One Health and transdisciplinarity discussions. JG and AA contributed to the Economics discussions. AA and DE contributed to the Anthropology discussions. All authors contributed to the writing of the manuscript, read, edited, and approved the final manuscript.

## Funding

Publication fee for this article was granted by a scholarship received from an anonymous donor of One Health Brasil.

## Conflict of Interest

The authors declare that the research was conducted in the absence of any commercial or financial relationships that could be construed as a potential conflict of interest.

## Publisher's Note

All claims expressed in this article are solely those of the authors and do not necessarily represent those of their affiliated organizations, or those of the publisher, the editors and the reviewers. Any product that may be evaluated in this article, or claim that may be made by its manufacturer, is not guaranteed or endorsed by the publisher.

## Footnotes

^1^In a classic illustration in this regard, Viveiros de Castro stated that jaguars, seeing themselves as humans, see humans as tapirs or wild pigs, so for them, our blood can be beer. If humans start to see the blood of his relatives as beer, taking the perspective of jaguars, this means that we are being taken to a different world to turn into a jaguar.

^2^The term “uncommon” - as used here - comes from the discussions of the anthropologist Marisol de la Cadena (44). In this context, the expression refers to a status of what is not universally shared, that is, of what is not the same among worlds.

^3^According to the anthropologist Eduardo Viveiros de Castro, in the perspectivism theory, to control an equivocation means to listen and speak knowing that the referents are different. That is, the difference between terms must be communicated and controlled in the dialogue. In symmetric humanity, to control equivocations is a way to preserve life (45).

## Panel 1. Glossary

**Anthropocene:** It is an expression that designates a new era, subsequent to the Holocene, in which humanity has become a global geological force capable of changing the existence of systems and life forms on Earth (8).

**Challenging factors:** Important elements evidenced by the social sciences that, in a transdisciplinary context, increase the awareness of complexity of health topics. Such elements challenge the way health has been traditionally understood and practiced in the modern world. Challenging factors can be, for example: ontological differences; gender, racial or ethnic inequalities; religious or regional aspects (our definition).

**Equivocation:** Concept introduced by the anthropologist Eduardo Viveiros de Castro - in the Perspectivism theory - that seeks to explain the mode of communication between Amerindian inhabitants (human and non-human) in the Brazilian Amazon. The term refers to the “referential alterity between homonymic concepts”, or the “mode of communication par excellence between different perspectival positions” (43, 45). See more in section Anthropology: When Assumptions are not Common - or the Same - Among Worlds of this current article.

**Externality:** Benefits or costs arising from the production or consumption process that are not considered in the economic model (24).

**Multiple worlds (pluriverse):** Discussion that confronts the idea of modernity that there is only one world, proposing “a world of many worlds” or a world in which many different worlds can fit, such as the Indigenous and non-Indigenous worlds (62). See more in section Anthropology: When Assumptions are not Common - or the Same - Among Worlds of this current article.

**One Health:** A concept that acknowledge that the health of humans, animals, plants and ecosystems are deeply connected and therefore must be thought and worked together. For this, it is suggested the use of transdisciplinary and multisectoral health strategies and approaches (our definition).

**Ontology:** We use “ontology” as defined by Mario Blaser (2010) in three distinct – but not excluding – layers of meaning: 1. From sociology - “kinds of being and their relations”; 2. From science and technology - “ontologies are shaped through the practices and interactions of both human and non-humans;” and 3. From his ethnographic work - “ontologies manifest as “stories” in which the assumptions of what kinds of things and relations make up a given world are readily graspable” (42).

**Regimes of Truth:** Concept introduced by Michel Foucault in 1975 defined as the “types of discourse it [society] harbours and causes to function as true; the mechanisms and instances which enable one to distinguish true from false statements, the way in which each is sanctioned; the techniques and procedures which are valorised for obtaining truth; the status of those who are charged with saying what counts as true” (63).

**Transdisciplinarity:** as the prefix “trans” indicates, it concerns what is - at the same time - *between* disciplines, *trough* the different disciplines and *beyond* any discipline. The aim of transdisciplinarity is the understanding of the present world(s), for which one of the imperatives is the unity of knowledge (50).
